# Computational prediction and experimental validation of *Ciona intestinalis *microRNA genes

**DOI:** 10.1186/1471-2164-8-445

**Published:** 2007-11-29

**Authors:** Trina M Norden-Krichmar, Janette Holtz, Amy E Pasquinelli, Terry Gaasterland

**Affiliations:** 1 Scripps Institution of Oceanography, University of California, San Diego, 9500 Gilman Drive, MSC 0202, La Jolla, CA 92093 USA; 2 Department of Biology, University of California, San Diego, La Jolla, CA 92093 USA

## Abstract

**Background:**

This study reports the first collection of validated microRNA genes in the sea squirt, *Ciona intestinalis*. MicroRNAs are processed from hairpin precursors to ~22 nucleotide RNAs that base pair to target mRNAs and inhibit expression. As a member of the subphylum Urochordata (Tunicata) whose larval form has a notochord, the sea squirt is situated at the emergence of vertebrates, and therefore may provide information about the evolution of molecular regulators of early development.

**Results:**

In this study, computational methods were used to predict 14 microRNA gene families in *Ciona intestinalis*. The microRNA prediction algorithm utilizes configurable microRNA sequence conservation and stem-loop specificity parameters, grouping by miRNA family, and phylogenetic conservation to the related species, *Ciona savignyi*. The expression for 8, out of 9 attempted, of the putative microRNAs in the adult tissue of *Ciona intestinalis *was validated by Northern blot analyses. Additionally, a target prediction algorithm was implemented, which identified a high confidence list of 240 potential target genes. Over half of the predicted targets can be grouped into the gene ontology categories of metabolism, transport, regulation of transcription, and cell signaling.

**Conclusion:**

The computational techniques implemented in this study can be applied to other organisms and serve to increase the understanding of the origins of non-coding RNAs, embryological and cellular developmental pathways, and the mechanisms for microRNA-controlled gene regulatory networks.

## Background

Small non-coding RNA genes have emerged in recent years as regulators of transcription and translation in a time and cell-state dependent manner. In particular, mature microRNA (miRNA) molecules of approximately 22 nucleotides in length have been shown to inhibit gene expression by base pairing to target mRNA. MicroRNAs have been implicated in gene regulation during embryological development, disease, and cell differentiation [1]. Specifically, recent studies have linked miRNA to many diverse biological processes such as clearing of maternal mRNA during zebrafish embryogenesis [2], regulation of the life span in *Caenorhabditis elegans *[3], and involvement in tumorigenesis in human cancer [4].

Although miRNAs were first discovered in the early 1990's, it has only been in the past five years that there has been an explosion in the literature on their formation and gene regulatory mechanisms, and on their abundance in the genomes of different organisms. MicroRNAs are now projected to occur at a frequency of approximately 0.5–1.5% of the total genes in the genome [5]. For the human genome, which contains approximately 30,000 protein coding and large non-coding RNA genes, there are currently 470 miRNAs reported in the miRNA database [6]. It is estimated that possibly 20 to 30% of human genes are targets of miRNA [7]. Since many miRNAs are evolutionarily conserved [8], discoveries in model organisms may contribute to understanding the role of specific miRNAs in gene regulatory networks for other organisms.

Recent studies have revealed many of the biological characteristics of miRNA formation and mechanism, which can be used for computational prediction of new miRNAs and their targets [9]. The microRNA primary transcripts (pri-miRNAs) contain one or multiple hairpin secondary structures. The ribonuclease Drosha complexes with a double-stranded RNA binding protein DGCR8, to process the pri-miRNA into a 70–100 nucleotide precursor miRNA (pre-miRNA). The pre-miRNA is exported from the nucleus to the cytoplasm via an Exportin-5 transport mechanism. Once in the cytoplasm, the RNase III enzyme Dicer cleaves the loop of the hairpin. The strands are separated, allowing the single-stranded mature microRNA whose 5'end has lower stability base pairings, to associate with the RNA-induced silencing complex (RISC), while the other strand is degraded. In RISC, the miRNA acts as a guide to recognize mRNA targets via base pairing. The mechanism of regulation is not entirely known, but the current model suggests that if the base pairing of the mature miRNA in the RISC complex shows sufficient complementarity to the mRNA target, then there will be cleavage and degradation of the mRNA target. With less complementarity, the miRNA/RISC represses translation of the mRNA target [10].

For animal miRNAs that partially base pair to target sequences, several general characteristics further constrain miRNA target prediction [10]. First, the miRNA binds to the 3' untranslated region (UTR) in most of the established mRNA targets in metazoans. Second, the strongest base-pairing between the miRNA and mRNA seems to occur at the 5' end of the miRNA, especially in the first 8 or 9 nucleotides. Third, sequence conservation in the UTRs of orthologous genes is a strong indicator of functional binding sites, and reduces false positive targets. Fourth, miRNA target regulation has been shown to occur when the same miRNA can recognize multiple sites in the 3'UTR of an mRNA target. Recently, multiple miRNAs in the same family, i.e., mir-48, mir-84, and mir-241 in *C.elegans*, were found to act in concert to control developmental genes [11]. Accurate target prediction must consider all of these properties of miRNA. Target prediction is difficult because of the very short length of the mature miRNA and the imperfect binding to the target. A variety of methods have been reported in the literature for the computational prediction of microRNA sequences and their targets [12].

This study involves the combination of computational and biological techniques to identify and validate microRNAs in the sea squirt, *Ciona intestinalis*. We performed these analyses in *C. intestinalis *for several reasons. First, as a member of the subphylum Urochordata (also known as Tunicata) whose larval form has a notochord, the sea squirt is situated phylogenetically at the emergence of the vertebrates [13], and therefore can provide information about the evolution of molecular regulators of early development. Second, the *C. intestinalis *genome has been completely sequenced [14], and another related urochordate genome, *Ciona savignyi*, has also been sequenced. Third, it has a compact genome of approximately 160 megabases (Mb), containing few repeated sequences. This small size and the consequent optimization of intergenic regions makes the genome tractable to study computationally. Fourth, *Ciona *is relatively easy to obtain and culture, and embryos can be manipulated experimentally with genetic techniques [15-17].

While another group has computationally predicted microRNAs for *Ciona intestinalis *[18], there are currently no experimentally validated microRNAs for *Ciona intestinalis *in the Sanger microRNA database, miRBase [6] (formerly known as "The microRNA Registry" [19]). To qualify for inclusion into the miRBase database, the expression of the microRNA must be detected using either the Northern blotting method, or from a library of cDNA made from size-fractionated RNA [20].

In the present study, we implemented microRNA gene and target prediction techniques to examine the genomes of the marine organisms *Ciona intestinalis *and *Ciona savignyi*. In our parameterized approach, we capitalized on the phylogenetic conservation of known miRNA gene families to screen quickly the genome of an organism for the possible presence of homologous miRNA genes. This technique greatly reduces the number of putative miRNAs that must be validated experimentally. Using the current approach, we predicted 14 miRNA sequences for *Ciona intestinalis*. We validated 8, out of 9 attempted, of the predicted miRNA sequences by Northern blot experiments. Following the prediction of the miRNA sequences, we computationally predicted and tabulated the most probable mRNA targets for these miRNAs. A subset of these targets exhibit conservation across phylogeny [21], and therefore may point to microRNA-controlled regulatory functions across evolutionary pathways.

## Results and Discussion

### Collection of known miRNA statistics

To tune the miRNA prediction algorithm parameters, miRNA and precursor sequences were analyzed to determine physical and sequence conservation characteristics, using the following three pairs of organisms: *Caenorhabditis elegans *vs. *Caenorhabditis briggsae*, *Drosophila melanogaster *vs. *Drosophila pseudoobscura*, and *Homo sapiens *vs. *Pan troglodytes*. Additional File 1 summarizes the statistics gathered for these three pairs of closely related organisms. For each species examined, the average percent identity of the two hairpin stem sequences was 78% or better, with a minimum of 65% identity. The average percent identity of the mature miRNA sequence between closely related species was 98%. The average hairpin loop length was 20 nucleotides, and ranged from 7 to 58 nucleotides. The percent GC content in the mature and precursor sequences was approximately 45%. These characteristics directed our microRNA prediction algorithm.

### Computational prediction of the *Ciona intestinalis *microRNAs

**Figure 1:**
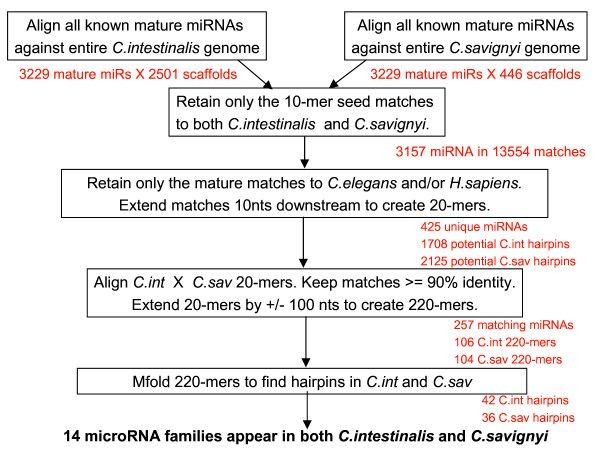
Flow diagram for microRNA prediction algorithm.

**Figure 2:**
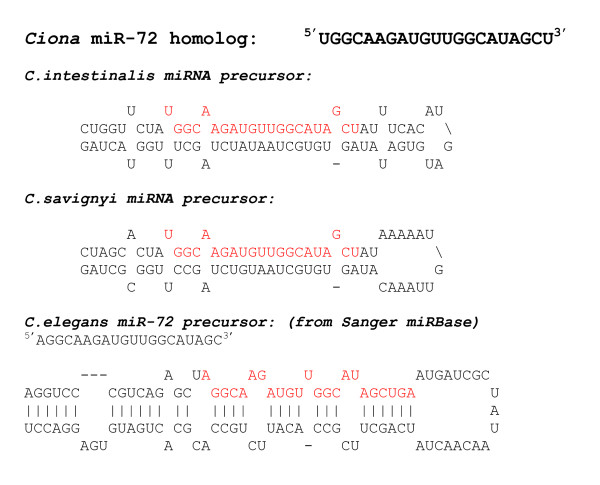
**RNA folding structure as calculated by the program *mfold *for miR-72**. Text output of putative miR-72 for *Ciona intestinalis*, *Ciona savignyi *and *C.elegans*.

**Figure 3:**
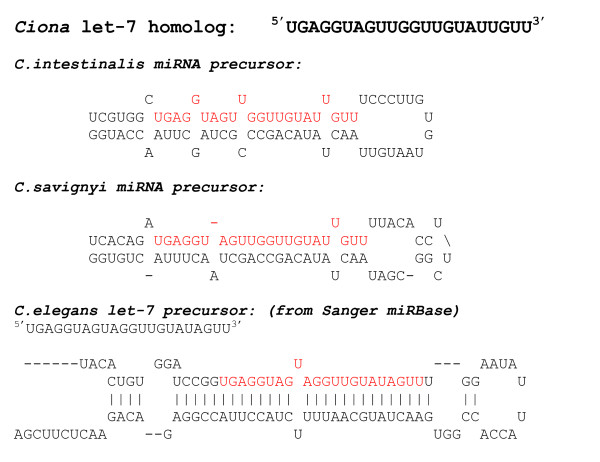
**RNA folding structure as calculated by the program *mfold *for let-7**. Text output of putative let-7 for *Ciona intestinalis*, *Ciona savignyi *and *C.elegans*.

**Figure 4:**
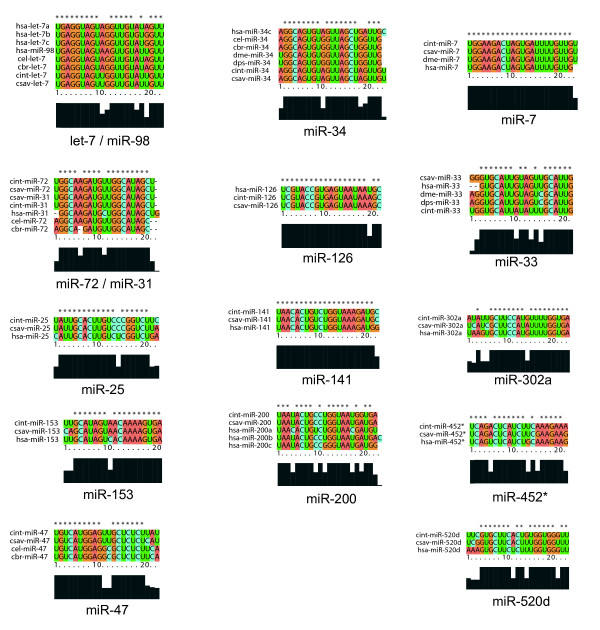
**ClustalX alignments of the miRNA predictions**. The first two columns contain the alignments for the miRNAs that we attempted to validate. The third column contains the alignments for the five miRNAs that we did not attempt to validate.

Using the parameters from the statistical analysis (Figure 1), we predicted 14 miRNAs for *Ciona intestinalis*. Mapping the locations of these putative *Ciona *miRNAs to the *Ciona *genome yielded additional information for comparison to the available known miRNAs. Additional File 2 lists the putative *C. intestinalis *miRNAs, their mature miRNA sequence, oligonucleotide probe sequence, scaffold number, coordinates, strand, position, and nearest neighboring gene. Figures 2 and 3 show the *mfold *program's text and graphical output for the putative miR-72 and let-7 for *Ciona *and *C. elegans*. The *mfold *output demonstrates the conservation of the precursor hairpin structure with the related species, *Ciona savignyi*. Using the ClustalX program, the predicted mature miRNA sequences for *C. intestinalis *and *C. savignyi *were aligned with known mature miRNA sequences from other organisms. Figure 4 shows the mature miRNA multiple sequence alignments which in turn demonstrate the membership of the predicted *Ciona *miRNAs in existing miRNA gene families.

**Figure 5:**
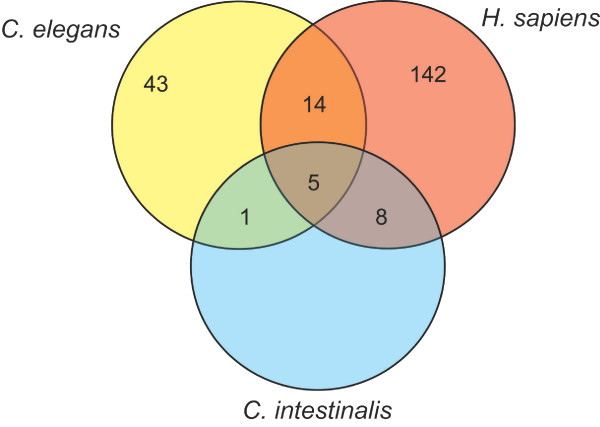
**Venn diagram summarizing the distribution of the predicted *C. intestinalis *miRNAs into the *C. elegans *vs. *H. sapiens *families**. Family counts for *C. elegans*, and for the intersection of C. elegans with *H. sapiens *were based on conserved 6-mer seeds [22]. The family count for *H. sapiens *was extracted from the miFam.dat microRNA family data file available from the miRNA database [6].

The number of miRNAs from this computational prediction is likely to be lower than the actual number of miRNA genes in *C. intestinalis*. Since miRNAs are believed to occur at a frequency of approximately 0.5–1.5% of the total genes in the genome [5], *Ciona*'s 15,000 genes should have generated between 75 – 225 miRNAs. Of the 19 miRNA families that appear in both *C. elegans *and *H. sapiens *[22], our algorithm only predicted 5 of these families in *C. intestinalis *(Figure 5). We expected to find all of the miRNA families that were present in both *C. elegans *and *H. sapiens*. Therefore, this may be an indication that our algorithm is underpredicting the miRNAs. The parameters of our prediction algorithm could be relaxed in future studies, in order to produce additional miRNA sequence candidates.

### Experimental validation of predicted miRNA sequences

**Figure 6:**
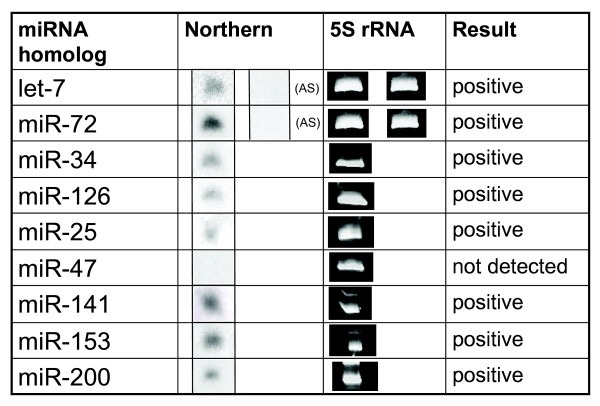
**PAGE Northern blot validation of miRNA predictions**. PAGE Northern blot analyses using adult *C. intestinalis *total RNA were performed to determine the indicated miRNAs. Ethidium bromide staining of the 5S rRNA is shown as a control for RNA loading and quality. Anti-sense (AS) probes were tested for let-7 and miR-72.

We experimentally validated 8, out of 9 attempted, of the putative *C. intestinalis *miRNA sequences using Northern blot analysis (Figure 6). To validate the strand polarity of the predicted mature miRNAs, we performed the Northern blot analysis with sense and anti-sense probes for the top and bottom strands of the let-7 and miR-72 *C. intestinalis *homolog predictions. In both the let-7 and miR-72 homologs, no hybridization to the anti-sense strand occurred. Therefore, the strand polarity of these two predicted miRNA sequences was confirmed.

As a control, equal quantities of total RNA from *C. elegans *and *C. intestinalis *were run on the same Northern blot [results not shown]. When hybridizing against the probes for the miR-72 and let-7 *Ciona *homologs, the *C. intestinalis *lanes showed strong positive signals. The *C. elegans *lanes showed a weak response to the *Ciona *miR-72 homolog probe, and no response to the *Ciona *let-7 homolog probe. These results were as expected, since Northerns are extremely sensitive to the probe sequence. Since there were known mismatches to the probe at the ends of the *C. elegans *sequence, it did not hybridize as well to the miR-72 probe as *C. intestinalis*. Similarly, since the *Ciona *let-7 sequence contained 2 alignment gaps in the middle section the *C. elegans *sequence, we did not expect to see hybridization.

Hybridizations with 7 of the remaining predicted *C. intestinalis *miRNAs were attempted, yielding 6 additional positive Northern blot results. For these putative miRNA sequences, probes were made against the predicted mature miRNA sequences only, not against the bottom sequences of the hairpin structure.

MicroRNA expression data from other organisms confirmed the results that we obtained in the Northern analyses of our predicted *C. intestinalis *miRNA sequences [22-26]. For example, let-7 is highly expressed in adult tissue of *C. elegans*, *D. melanogaster*, and vertebrates [23]. Therefore, we were not surprised to find the let-7 homolog expressed in the adult tissue of *C. intestinalis*. Conversely, miR-47 has been experimentally validated in *C. elegans*, but has not yet been predicted or validated in vertebrates [22]. Although computationally there appears to be a miR-47 homolog in *C. intestinalis*, we were not able to detect its expression by Northern analysis. Since *C. intestinalis *is phylogenetically located at the emergence of vertebrates, the expression of miR-47 in *C. intestinalis *may be more like vertebrates in this case.

We chose not to validate our predictions of miR-302a, miR-33, miR-452*, miR-520d, and miR-7, based upon reports of the expression of these homologs in the literature. The miR-302a sequence was validated by others in mouse and human by cloning from embryonic stem cells. Likewise, the miR-7 sequence was found in early embryonic development of the Drosophila (embryo to 6 hours) [23]. Our validation was done with adult tissue. The miR-452* and miR-520d sequences were detected by the array-cloning technique [27]. The miR-33 sequence failed to validate by Northern in any of the tissues tested in a previous study, which included HeLa cells, mouse kidney, adult fish, frog ovary, and S2 cells [23]. Therefore, since it was unlikely to detect these homologs with Northern analyses in adult tissue, we have not attempted to validate them at this time.

### Computational prediction of the *Ciona intestinalis *mRNA targets

**Figure 7:**
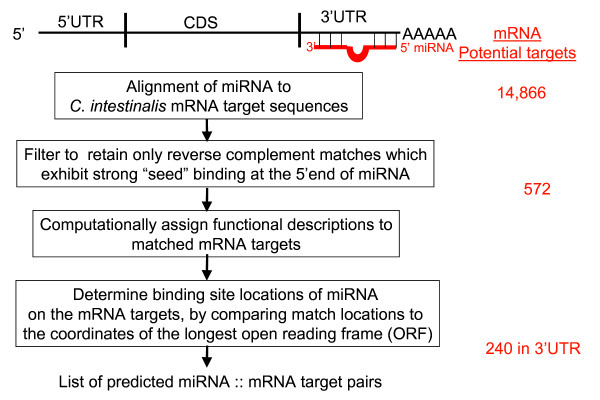
High level flow diagram of mRNA target prediction algorithm.

Application of the target prediction algorithm allowed stepwise refinement of the 14,866 potential mRNA targets to a high confidence list for validation. The algorithm for mRNA target prediction was based upon the observed biological mechanism of miRNA in the regulation of gene expression. That is, the target prediction algorithm addresses the following miRNA properties: reverse complementary partial binding of the miRNA to the target; the most critical binding at the 5' end of the miRNA strand; and the binding to the 3'UTR of the target. Figure 7 contains a summary of the results of the computational target prediction. The 14,866 mRNA sequences were input to the prediction pipeline as potential targets for the *Ciona *miRNA. The Smith-Waterman alignment algorithm, highly sensitive to small sequences, reduced the number of potential targets to 572 unique mRNAs. These sequences were filtered to retain only reverse complementary matches in which the miRNA 5'end had the strongest affinity for the target.

**Figure 8:**
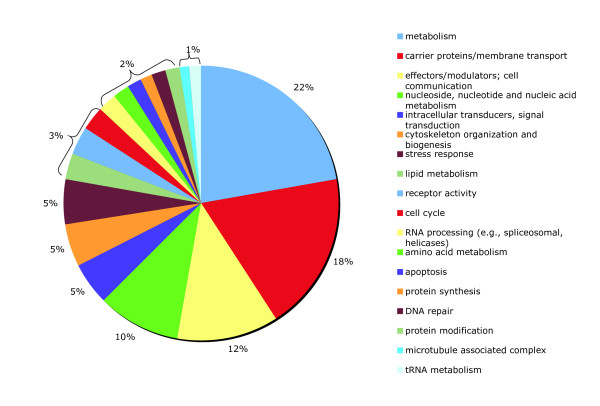
Gene ontology (GO) terms grouping of the mRNA targets.

If 20 to 30% of the genes for an organism are regulated by miRNA, then *Ciona intestinalis *should have approximately 3000 to 4500 mRNA targets based upon its 15,000 gene size. However, since our algorithm underpredicted the number of *Ciona *miRNAs by approximately a factor of 10, this would explain a corresponding reduction of predicted targets. Therefore, 240 targets for 14 gene families is a reasonable number of possible targets. Preliminary experimentation with the program parameters demonstrated that varying the seed alignment lengths and sequence identity percentages reflected a direct correlation to the number of predicted targets. Configuring these parameters may then be used to adjust the system to produce more or less targets. Through computational analysis, we assigned functional descriptions to the target genes which had miRNA matches to the 3'UTR of the mRNAs. The target genes were also grouped according to their Gene Ontology (GO) terms (Figure 8). The majority of target gene functions are involved in metabolism, membrane transport, and cell signaling.

To enforce the relationship between the miRNA binding location and its potential to elicit a regulatory effect in the target gene, we computationally calculated and assigned binding locations to each target. The results were tabulated and sorted according to the mRNA target match locations occurring in the 3' untranslated region (3'UTR), 5' untranslated region (5'UTR), and protein coding section (CDS). This procedure divided the 572 target matches of the *Ciona *mRNA sequences into the following subsets: 240 matches to the 3'UTR, 262 matches to the 5'UTR, and 70 matches to the CDS. As most established miRNA targets exhibit binding in the 3'UTR [28], this region was examined more closely. Further manual curation yielded three instances of multiple hits to the 3'UTR of the same target by different miRNAs. Additional File 3 contains a list of the predicted targets with single and multiple hits to the 3'UTR region. The predicted targets which contained multiple hits would be interesting to validate because there is evidence in other organisms that multiple miRNA binding sites in the 3'UTR contribute to more potent regulation [11,29].

Finally, we attempted to use phylogenetic homology with the *C. savignyi *genome to further prune the list of targets. There are no functional gene descriptions available for *C. savignyi*, and surprisingly, the *C. intestinalis *and *C. savignyi *genomes are more divergent than we had expected. Therefore, this step removed virtually all of the potential targets, and had to be abandoned from our algorithm. Other studies have also found that approximately 50% of the targets would have been missed if conservation had been used [2].

**Figure 9:**
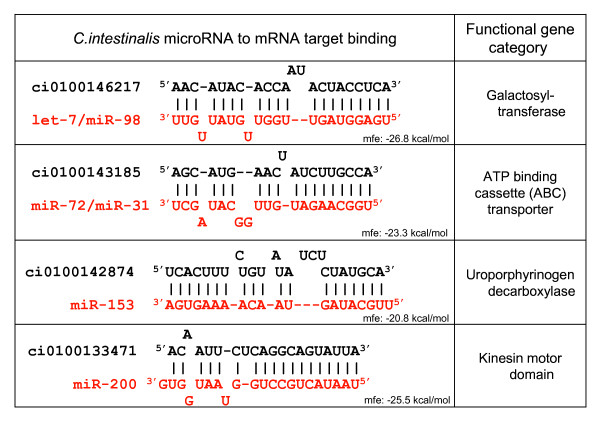
**Sampling of predicted mRNA targets**. ("mfe" is the minimal free energy of the duplex, as calculated by RNAhybrid.)

Because we could not use phylogenetic conservation with *C. savignyi *for the final step, we assessed whether other features supported the strength of the microRNA to mRNA target pairings. That is, we evaluated seed region match quality; we excluded G:U wobble pairs; and we examined whether some amount of compensatory binding at the 3' end of the miRNA was present. Figure 9 shows a sampling of the predicted mRNA targets. The RNAhybrid program [30] was applied to these miRNA:mRNA sequence pairs, to calculate the minimal free energy of the hybridization. The values obtained from RNAhybrid were consistent with energetically favorable hybridizations. Notably, for approximately 25% of the predicted targets, these functional gene orthologs were also predicted for human/mouse/rat using the PicTar target prediction program [21].

## Conclusion

In this study, we used computational methods to predict 14 miRNA sequences in the *Ciona intestinalis *genome. Of the 9 miRNA sequences tested experimentally using Northern blot analyses, we successfully validated the presence of 8 of our predicted *Ciona *miRNA homologs in the RNA of the adult tissue of *Ciona intestinalis*. Currently, no experimentally validated miRNAs are reported in the Sanger miRBase for *Ciona intestinalis*. The addition of our eight validated sequences to the database strengthens the evidence of phylogenetic miRNA sequence conservation. Additionally, a computational algorithm for mRNA target prediction was developed in this study, which takes advantage of the known biological properties of miRNA function and their target binding behavior.

Although previous studies have used phylogenetic homology for the prediction of miRNAs, our algorithm involved several novel concepts. First, since *Ciona *is located in an area of the phylogenetic tree for which miRNAs have not yet been validated, we chose to search for potential miRNA sequences across widely divergent phyla and subphyla. In other homology-driven approaches, the organism under study could be examined for known miRNAs from a member of the same Class or Order [31-33]. Second, in the initial step of our algorithm, the strongest constraint for alignment was placed upon the first 10 nucleotides, which includes the seed region of the miRNA. This differs from other methods that searched along the entire mature miRNA for perfect or near-perfect matches [12,34-36]. This constraint was chosen because the seed region is important in target binding [7], and has been used in other studies as the basis for determining miRNA family membership [22]. Third, our algorithm used a second homology filter to compare the miRNA candidates between two species for which miRNAs have not yet been validated. Because the two genomes of *Ciona *are so divergent, these conserved ~22 nucleotide sequences are even more compelling as potentially valid miRNA genes.

In the course of this study, another laboratory published an initial computational prediction of miRNAs for *Ciona intestinalis *[18], based upon homology between predicted precursor hairpin structures in *Ciona intestinalis, Ciona savignyi*, and *Oikopleura dioica*. Their published prediction of 41 miRNA precursor sequences overlapped with ours by just one microRNA, let-7. In a subsequent study [37], a clustering approach was used with the *Ciona *non-coding RNA candidate sequences. By this method, the authors predicted 58 miRNAs, of which only let-7, miR-7, miR-124, and miR-126 coincided with known miRNAs. Our method predicted let-7, miR-7, and miR-126, as well as 11 other conserved miRNA families. While their approach exhibits great potential for predicting conserved and novel miRNA candidates, they report no experimental validation in either study, so we cannot evaluate the accuracy of their prediction method's results.

Our miRNA prediction code was written to be highly configurable, allowing for different binding strengths, as well as step-wise refinement to prune the candidates by phylogenetic conservation with homologous miRNA gene families. The entire prediction pipeline was designed so that intermediate results could be extracted at each step and examined for interesting patterns. As more characteristics of miRNA are discovered, code modules can be added and removed to reflect the changing paradigm. Establishing statistics for the characteristics of known miRNA genes in other organisms enabled the creation of a parameterized prediction approach with a high success rate. Genomic sequence analysis for conserved miRNA genes, coupled with secondary structure folding, is not necessarily sufficient to find miRNA genes in an organism unless parameters are applied to aid in the selection process. In a recent study involving the prediction of porcine miRNA genes through homology, only 7 out of 20 attempted predictions were validated by Northern blot [31]. The details of the prediction algorithm in this study were not shown, so it is not clear if parameterization was applied during the process. Our code was also designed so that parameters could be configured to tighten or relax the constraints on the matches. The current parameters may be too restrictive, if the percentages reported in the literature [5] are extendable from human miRNAs to *Ciona *miRNAs. That is, we should have obtained approximately 75 to 225 miRNAs based upon the 15,000 gene size of the *Ciona intestinalis *genome. In future studies, several constraints could be relaxed in order to potentially predict additional miRNAs in *Ciona intestinalis*. In particular, the length of alignment and percent identity between the *C. intestinalis *and *C. savignyi *mature miRNA candidates could be reduced. Further, the requirement of structural similarity, involving a reasonable conservation of bulge size and location in the hairpin stem region of the predicted miRNA precursors, could be decreased between these two species of *Ciona*.

Additionally, some miRNA genes and their targets may have been missed by the phylogenetic conservation constraint to the *C. savignyi *genome. Our inability to find orthologous targets across *C. intestinalis *and *C. savignyi *may be due to several factors. First, the *C. savignyi *genome has ambiguous and incomplete regions, which prevented sufficient alignment with the *C. intestinalis *genome. Second, the two species of *Ciona *have recently been found to be far more divergent than expected for such morphologically similar species. Using an analysis of 18S rRNA sequences, it has been estimated that "the divergence between the two species of *Ciona *is slightly greater than that between human and chick [38]." Additionally, the *Ciona *species exhibit an extremely high allelic polymorphism. The allelic polymorphism for *C. intestinalis *has been estimated to be an average of 1.2% [13]. Sequencing of two haplotypes of *C. savignyi *had an extremely high heterozygosity rate of approximately 4.6% [39]. Because of the high polymorphic rate, the *C. savignyi *genome could not be assembled using the classical whole-genome assembly method. Instead, two haplotypes were sequenced separately, and then merged to produce the reference sequence. To confirm our predictions in *C. savignyi*, it would be necessary to examine the pre-assembled alleles for the sequence. Therefore, it was necessary to reevaluate our target prediction algorithm and remove the constraint of phylogenetic conservation with *C. savignyi*, in order to produce a larger sample set for validation.

Despite these bioinformatic challenges, our miRNA prediction success rate demonstrates that this algorithm may be used for other organisms, including those with poorly or unannotated genomes. Further, our study highlights the benefit of the selection of *C. intestinalis *as a model organism for miRNA experimentation. In particular, since miRNA expression appears to be tissue-specific, many miRNAs of higher vertebrates might not be detected due to the specific tissue or organ that is sampled. In *Ciona*, the entire adult organism, including all organs, can be homogenized and prepared for RNA extraction. Therefore, all tissue types of *Ciona *are represented in the homogenate that is used for miRNA detection, thereby increasing the probability of successful validation.

A disadvantage to our miRNA prediction method is that it will not find novel miRNAs in a genome. However, the information of the existence of known ones in different species may be used to refine the method of finding novel ones. In the genome of an organism, one can find many hairpin loops, and short conserved sequences as potential miRNA candidates. However, not all of these candidates will produce a mature miRNA. Therefore, as more miRNAs are experimentally confirmed, refinements can be applied to the prediction algorithms.

Our computational predictions could also be used to construct a gene regulatory network model, using hierarchical clustering of the miRNA and mRNA target binding strengths. Initial experimental gene regulatory networks are being constructed at several laboratories for *Ciona intestinalis *[40,41]. However, there are currently no published miRNA-based regulatory networks for *Ciona intestinalis*. The development of a computational gene regulatory network model of miRNA to target mRNA mappings for *Ciona *would facilitate the process of experimental validation and gene regulatory mechanism discovery.

The outcome of this study involves the implementation of computational techniques which can be applied to the study of other organisms. In particular, the technique can be used to quickly screen the genome of any organism for the presence of existing miRNA homologs. The prediction and validation of these factors may increase the understanding of evolution of microRNA-controlled regulatory relationships and give insight into the origins of microRNA networks in diverse animal species.

## Methods

### Collection of known miRNA statistics

The analysis of characteristics of known miRNAs for several species yielded parameters used to predict miRNAs and their mRNA targets in the two *Ciona *genomes. Mature miRNA and precursor sequences were obtained from the Sanger Institute's miRBase database ([6], July 2006). The statistics collected for these known miRNA molecules included loop length, percent identity in the stem region of the hairpin structure of each miRNA, percent identity of the mature miRNA sequences between the two closely related species, and percent GC content of the sequences.

### Computational prediction of the *Ciona intestinalis *microRNAs

Computational prediction of *Ciona intestinalis *and *Ciona savignyi *microRNAs was performed with genome data available from the Department of Energy Joint Genome Institute (JGI) and the Broad Institute. The *Ciona intestinalis *genome, Assembly v1.0 (April 2002) and Annotation (V1.0), were obtained from the JGI website [14]. The *Ciona savignyi *genome, Assembly v1.0 (April 2003), was obtained from the Broad Institute website [42]. Using statistics for characteristics of known microRNAs gathered in the first phase of this study, a miRNA prediction algorithm was implemented to search the *Ciona intestinalis *genome for conserved mature microRNA genes (Figure 1).

The entire *C. intestinalis *and *C. savignyi *genomes were searched for conservation with the seed region of the known mature miRNA sequences from the Sanger miRBase. Although it has been estimated that 60% of miRNA primary transcripts are found in the intergenic regions [43], we chose to search the entire genome to avoid the omission of any miRNA genes that might occur in introns or coding regions. The list of mature miRNA sequences for all organisms was obtained and used as query sequences. Queries were aligned locally with the target genomes using the FASTA/ssearch34 program [44]. Software was written to examine the results and extract matches of high similarity. Matches of 90% identity or better to the first 10 nucleotides of the known miRNAs were retained for further processing. Conservation of 90% identity or better in this seed region, between *C. intestinalis *and *C. savignyi*, was enforced. To remove repeated matches to miRNA gene family members, only the matches to *C. elegans *and/or *H. sapiens *were retained. The rationale for this choice was the following: if the sequences existed in *C.elegans *and/or *H.sapiens*, they were likely to exist in *Ciona *as well. To prune the number of potential hairpin structures, the list of seed matches between *C. intestinalis *and *C. savignyi *were extended by 10 nucleotides downstream from the match. These 20-mers were searched for 90% identity between the two species. For all such conserved matches, 100 nucleotides upstream and downstream from the match boundaries were extracted from the genomes and examined for a hairpin structure.

The RNA folding software *mfold *[45] was used to confirm the hairpin structure as the lowest energy folding form. The low energy state is indicative of the secondary structure that the RNA sequence is most likely to adopt. The structures were manually curated for the presence of hairpins with the mature miRNA sequence in the stem region, loop lengths between 10 and 50 nucleotides, and reasonable conservation of bulge size and location in the hairpin stem region between the two species. The procedure resulted in the prediction of 18 miRNA molecules that appear in both *C. intestinalis *and *C. savignyi*.

The ClustalX version 1.83 program [46] was used to confirm the miRNA gene family membership, and to verify homology to *C. elegans *and/or *H. sapiens*. ClustalX analysis yielded 14 miRNA gene families for *Ciona intestinalis*.

The genome browsing capability of the Satoh laboratory web site was utilized to examine the JGI Gene V1 and the Kyoto Grailexp Gene 2005 tracks of the *Ciona intestinalis *genome [42]. The coordinates for the putative *Ciona *miRNAs were mapped to the gene locations to determine the position and nearest neighboring genes.

### Experimental validation of predicted miRNA sequences

We chose nine sequences from our list of 14 predicted miRNAs for experimental validation. The validation process involved the collection, culturing, dissection, and RNA extraction from adult *Ciona intestinalis*. The total RNA was analyzed via the Northern blot protocol with end-labeled DNA oligonucleotide probes, specific to our predicted miRNA sequences.

Adult *Ciona intestinalis *were collected in Mission Bay, San Diego, CA. The adults were cultured at Scripps Institute of Oceanography in an aquarium of constantly flowing filtered seawater, which was pumped from approximately 1000 feet offshore. The *C. intestinalis *were kept under constant light conditions to suppress the release of gametes.

Prior to RNA extraction, the tunic of the *C. intestinalis *was removed to avoid the inclusion of contaminating organisms and material that may be growing on the surface. The fresh tissue of the entire organism, without the tunic, was immediately homogenized in the Trizol reagent (Invitrogen), and a total RNA extraction protocol was performed.

We used polyacrylamide gel electrophoresis (PAGE) Northern blot methods to detect the presence of miRNA in the RNA samples [8,47]. For a positive control on some of the Northern blots, an equal amount of total RNA from the nematode *Caenorhabditis elegans *was loaded in adjacent lanes to the *C. intestinalis *RNA. The *C. elegans *RNA was extracted from wild-type N2 grown at 20C, and collected at 53 hours in young adult stage. This stage of *C.elegans *may contain some fertilized oocytes.

DNA oligonucleotides (Allele Biotechnology, Inc. and Integrated DNA Technologies, Inc.) were designed with reverse complementary sequence to the putative mature miRNA sequences. For the predicted let-7 and miR-72 *Ciona *miRNA homologs, oligonucleotides were designed as probes for both the top and bottom strands of the hairpin structure. For all predicted miRNAs, the probe was designed by extending the mature miRNA sequence by 3 nucleotides on each end. Additional File 2 lists mature predicted miRNA sequences and their corresponding probes.

### Computational prediction of the *Ciona intestinalis *mRNA targets

Following the prediction of the miRNA sequences, we computationally predicted and tabulated the most probable mRNA targets for these miRNAs. Figure 7 contains a flow diagram for the mRNA target prediction algorithm. The process aligns miRNA sequences against potential target sequences, filters the output data according to observed miRNA target binding characteristics, assigns gene functional descriptions, determines the binding locations, and prunes by phylogenetic homology with *C. savignyi*. Each individual step will be discussed in more detail in subsequent sections.

The Smith-Waterman algorithm performs a local alignment, as opposed to a global alignment. Although, it is more time-consuming, local alignment is more sensitive to finding smaller query sequences in the target sequences. A series of filtering and ranking algorithms were implemented in the Perl programming language. The procedure was written such that parameters could easily be modified and was based upon slight variations in a previously reported target prediction procedure [7] which capitalizes on the seed region binding tendencies. In particular, the output matches from the Smith-Waterman alignment were passed through a filter to confirm that the miRNA exhibited reverse complementary binding to the target. The matches were then classified according to the length of their seed binding as follows: *6-mer *matches with perfect complementary for nucleotides 2 – 7 of the 5' end of the miRNA; *7-mer *matches at nucleotides 2 – 8; *7-mer *matches at nucleotides 1 – 7; *8-mer *matches at nucleotides 2 – 9; and *8-mer *matches located at nucleotides 1 – 8. Our algorithm did not enforce the presence of an adenosine (A) on either side of the *6-mer *seed match.

The filtered matches were then ranked in two ways: by number of matches of miRNA for each mRNA, and by number of matches of mRNA per miRNA. The locations on the mRNA target were retained for processing of the relative hit location and for conservation with *C. savignyi*. These steps are will be elaborated in the next four paragraphs.

The potential target gene data set used for the target prediction was constructed as follows. Predicted gene transcripts for *Ciona intestinalis *(Annotation, V1.0) were obtained from the Satoh laboratory web site [48]. These predicted mRNA sequences did not necessarily contain the 5'UTR and 3'UTR regions of the mRNA. Thus, 1500 nucleotides upstream and downstream from the transcript boundaries were extracted from the *C. intestinalis *genome. We focused on matches to the 3'UTR of the target genes as the most likely genes regulated by miRNA.

Because the *Ciona intestinalis *genome is incompletely annotated, we computationally assigned functional descriptions to the sequences. The functional descriptions for most transcription factor genes and some signaling molecules, based on cDNA libraries and *in situ *hybridization studies, are available from the *Ciona intestinalis *online database [49]. However, many of the predicted transcripts across the genome were not functionally annotated. Therefore, the available data files were augmented with functional descriptions to the mRNAs based on blast alignments to proteins from the non-redundant database, "nr" [50], using the MESH component of MAGPIE [51]. The top match for each mRNA target was chosen as a surrogate functional description. The mRNA functional description list generated by MESH was joined to the InterPro (IPR) functional descriptions from the *Ciona intestinalis *online database, to produce a list of mRNA identifiers and their functional descriptions.

The Gene Ontology (GO) terms for the mRNA targets were assigned via custom software following alignment of the mRNA sequences with the GO database. The GO identifiers were then input into the Gene Ontology (GO) Terms Classification Counter [52], using the EGAD2GO classification filter for higher level grouping.

To further restrict the number of potential targets, the location of each binding site of a miRNA to a target gene was classified by location: 5'UTR, CDS, or 3'UTR. Matches to the 3'UTR of the mRNA were retained as the most probable targets of miRNA regulation. The EMBOSS *transeq *program [53] was used to translate the *C. intestinalis *mRNAs into 6 possible reading frames. A Perl script was implemented to select the longest open reading frame (ORF) and tabulate its coordinates in the mRNA sequence. The region of the sequence upstream of the ORF was assumed to be the 5'UTR, and the region downstream of the stop codon of the ORF was assumed to be the 3'UTR. The ORF itself was classified as the coding sequence (CDS). Next, another Perl script was written to compare the location of the miRNA match in the mRNA sequence, to the locations of the 5'UTR, CDS, and 3'UTR. Each miRNA match was classified as a 5'UTR, CDS, or 3'UTR match. This information was applied to the list of potential targets, which could then be sorted and ranked by the hit location.

The final step in the mRNA target prediction process examined the mRNA targets for conservation in *C. savignyi*. Currently, predicted transcripts of *C. savignyi *do not exist. *C.savignyi *"pseudo" mRNAs were generated as follows. The tera-tblastn program on the TimeLogic board aligned the *C.intestinalis *mRNA gene sequences against the entire *C. savignyi *genome. The top match of the *C. savignyi *genome to each of the mRNA sequences was classified as an orthologous *C. savignyi *mRNA. The target prediction code was applied to the *C. savignyi *subsequences, to check that miRNAs matched the 3'UTR of orthologous genes to *C. intestinalis*, according to the seed match criteria described earlier.

## Authors' contributions

TMNK designed and implemented the algorithms, and participated in the experimental validation. JH participated in the experimental validation. AEP guided development of the algorithm, and oversaw the experimental work. TG contributed to the development of the algorithm, and oversaw the project. All authors read and approved the final manuscript.

## Supplementary Material

Additional File 1 Analysis of the characteristics of known miRNAs. This file summarizes the miRNA statistics gathered for three pairs of closely related organisms: *Caenorhabditis elegans *vs. *Caenorhabditis briggsae*, *Drosophila melanogaster *vs. *Drosophila pseudoobscura*, and *Homo sapiens *vs. *Pan troglodytes*. Click here for file

Additional File 2 List of predicted miRNAs for *C. intestinalis *and their corresponding probe sequences. This file lists the putative *C. intestinalis *miRNAs, their mature miRNA sequence, oligonucleotide probe sequence, scaffold number, coordinates, strand, position, and nearest neighboring gene. Click here for file

Additional File 3 List of predicted miRNA targets for *C. intestinalis*. This file contains a list of the predicted targets with single and multiple hits to the 3'UTR region. Click here for file
