# Supplementary material for: Computational prediction and experimental validation of Ciona intestinalis microRNA genes
Source: BMC Genomics. 2007 Nov 29;8:445. doi: 10.1186/1471-2164-8-445 (PMC2243180; doi:10.1186/1471-2164-8-445)
Supplement: Additional File 1 — Analysis of the characteristics of known miRNAs. This file summarizes the miRNA statistics gathered for three pairs of closely related organisms: Caenorhabditis elegans vs. Caenorhabditis briggsae, Drosophila melanogaster vs. Drosophila pseudoobscura, and Homo sapiens vs. Pan troglodytes. [file 1471-2164-8-445-S1.pdf]

Organism	#miRs	Mean %Id In Stem	Max % Id in Stem	Min% Id in Stem	Mean loop length	Max loop len.	Min loop length	%GC	Mean % Id to close Org.	Max % Id to close Org.	Min% Id to close Org.	#mIR Match
**C.elegans**	105	76±4.6	89	65	21±6.7	51	7	45				
									96±5.6	100	80	78
**C.briggsae**	74	76±4.3	90	65	21±6.0	52	10	45				
**D.melano-gaster**	65	78±5.1	90	63	20±7.7	58	9	44				
									99.7±1.5	100	90	68
**D.pseudo-obscura**	58	78±5.1	87	66	20±6.3	46	9	43				
**H.sapiens**	262	80±5.6	100	64	18±4.4	42	7	46				
									100±0	100	100	74
**P.troglo-dytes**	74	80±5.3	94	70	17±3.6	35	10	46				
